# Supplementary material for: Identification of photoperiod-induced specific miRNAs in the adrenal glands of Sunite sheep (Ovis aries)
Source: Front Vet Sci. 2022 Jul 22;9:888207. doi: 10.3389/fvets.2022.888207 (PMC9354845; doi:10.3389/fvets.2022.888207)
Supplement: Supplementary file 1 [file Data_Sheet_1.ZIP › Supplementary file/miRDeep2 file.zip!/miRDeep2 file/Novel_Structure/SP42A2/Novel_295.pdf]

Provisional ID	: NC_019475.2_33214
Score total	: 13.1
Score for star read(s)	: 3.9
Score for read counts	: 6.2
Score for mfe	: 1.4
Score for randfold	: 1.6
Score for cons. seed	:
Total read count	: 24
Mature read count	: 14
Loop read count	: 0
Star read count	: 10

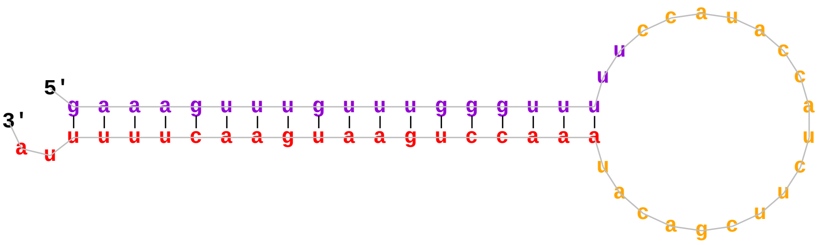

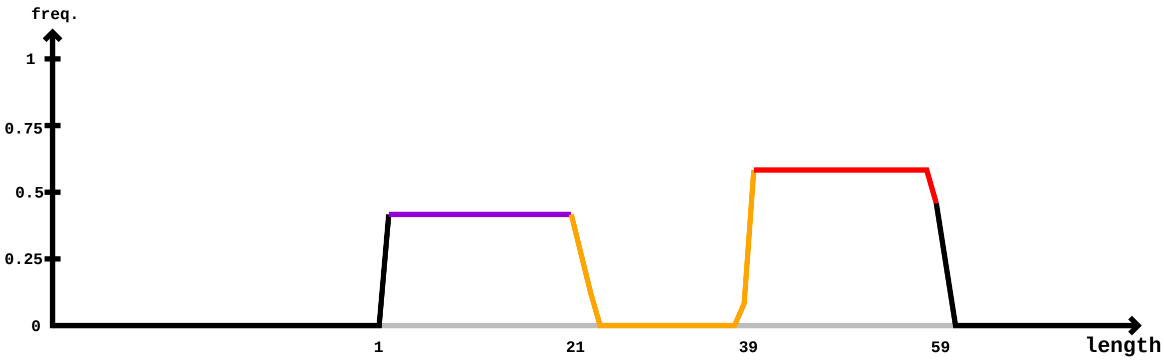


```
                                  Star                                 Mature
5'- auuuacaaagugaaggcuguauugggcuggccagaaaguuuguuuggguuuuuccauaccaucuucgacauaaaccugaaugaacuuuuuagccaacccaauaaaauacuc   -3'   obs
    auuuacaaagugaaggcuguauugggcuggccagaaaguuuguuuggguuuuuccauaccaucuucgacauaaaccugaaugaacuuuuuagccaacccaauaaaauacuc         exp
    ........(((....))).(((((((.((((.(((((((((((((((((((....................))))))))))))))))))).)))).)))))))........  reads  mm  sample
    .................................gaaaguuuguuuggguuuuu..........................................................  7      0   seq
    .................................gaaaguuuguuuggguuuuucA........................................................  3      1   seq
    ......................................................................Gaaaccugaaugaacuuuuu.....................  2      1   seq
    .......................................................................aaaccugaaugaacuuuuu.....................  1      0   seq
    .......................................................................aaaccCgaaugaacuuuuua....................  11     1   seq
```
